# Large Reduction in Postoperative Posterior Tibial Slope Risks Anterior Collapse of the Tibial Component in Fixed-Bearing Unicompartmental Knee Arthroplasty

**DOI:** 10.1016/j.artd.2022.06.011

**Published:** 2022-07-31

**Authors:** Shingo Kurihara, Kazuhisa Hatayama, Masanori Terauchi, Kenichi Saito, Hiroshi Higuchi, Hirotaka Chikuda

**Affiliations:** a Department of Orthopaedic Surgery, Gunma University Graduate School of Medicine, Maebashi, Gunma, Japan; b Department of Orthopaedic Surgery, Japan Community Health Care Organization Gunma Central Hospital, Maebashi, Gunma, Japan; c Department of Orthopaedic Sports Surgery, Asakura Sports Rehabilitation Clinic, Maebashi, Gunma, Japan

**Keywords:** Knee osteoarthritis, Unicompartmental knee arthroplasty, Posterior tibial slope, Anterior collapse, Postoperative knee joint function

## Abstract

**Background:**

Although the posterior tibial slope (PTS) of the tibial component in unicompartmental knee arthroplasty is recommended to be between 3° and 7°, variations in preoperative PTS are wide. The purpose of this study was to evaluate the influence of the changes in preoperative and postoperative PTS on clinical outcomes.

**Methods:**

One-hundred and eighty-two knees that underwent medial fixed-bearing unicompartmental knee arthroplasty were evaluated retrospectively. The mean follow-up period was 36.4 ± 13.2 months (range, 24 to 63 months). Preoperative and postoperative PTS were measured on lateral radiographs. Knees were classified in the large reduction group if the postoperative PTS was reduced by more than 5° compared with the preoperative value and in the small reduction group if not. Knee flexion angle and 2011 Knee Society Knee Scoring System were evaluated at the last follow-up of at least 2 years.

**Results:**

Thirty-three knees were classified in the large reduction group, and 149 knees were classified in the small reduction group. The preoperative and postoperative PTS of large and small reduction groups were 10.9 ± 2.2, 3.6 ± 2.4 degrees and 7.7 ± 2.7, 7.1 ± 2.4 degrees, respectively. Flexion angle and 2011 Knee Society Knee Scoring System were not significantly different between the groups. However, the incidence of anterior collapse of the tibial component in the large group was significantly higher than that in the other group (*P* < .001).

**Conclusions:**

Large reduction in the postoperative PTS may be associated with anterior tibial collapse, and therefore this study shows one potential benefit for matching native slope.

## Introduction

Unicompartmental knee arthroplasty (UKA) is a less-invasive alternative to total knee arthroplasty (TKA) for unicompartmental osteoarthritis or osteonecrosis. The number of UKA surgeries has been increasing because of its advantages in terms of faster recovery, lower morbidity, reduced blood loss, and better range of motion, function, and patient-reported outcomes (PROMs) than TKA [[1], [2], [3], [4], [5]]. However, UKA is a more technically demanding procedure, and the survival rate is inferior to that of TKA [6]. To achieve good clinical outcomes and durability after UKA, accurate implant placement and patient selection are important [7,8]. However, the optimal tibial component alignment remains a matter of debate.

Several authors reported that the optimal postoperative limb alignment is mild varus in the coronal plane to reduce the risk of disease progression in the lateral compartment and to achieve a good long-term clinical outcome [[8], [9], [10]]. Therefore, tibial component placement in slight varus has been accepted. However, tibial component placement in varus larger than 5° was reported to lead to loosening of the tibial component [11]. The posterior tibial slope (PTS) is also an important factor associated with knee kinematics and UKA durability. Hernigou and Deschamps [12] noted that a PTS of more than 7° should be avoided and recommended a PTS of between 3° and 7°. However, variations in the preoperative PTS are wide, and nearly half of preoperative tibial slopes are >7° [13,14]. To date, for knees with a larger PTS, there have been no studies on whether tibial component placement to reduce PTS has a risk of inferior clinical outcomes such as reduced postoperative flexion angle or pain.

The purpose of our study was to evaluate the influence of the preoperative and postoperative PTS changes on clinical outcomes, including PROMs. We hypothesized that knees with small postoperative PTS changes have better clinical outcomes, whereas those with a large reduction in PTS after surgery will have a reduced postoperative flexion angle.

## Material and methods

After approval by the institutional review board of the affiliated institutions, data were collected retrospectively. Between December 2015 and March 2020, 190 knees in 170 subjects underwent medial UKA for medial osteoarthritis or osteonecrosis at our institute. Seven patients (7 knees) were excluded because they did not complete 2 years of clinical follow-up. One patient (1 knee) died from an unrelated cause 1 year after surgery. Therefore, 182 knees in 162 subjects (61 male and 101 female) were included in this study. There was no cutoff of age in the UKA indication, and patellofemoral osteoarthritis was not excluded if the patient had no symptoms. In all cases, magnetic resonance imaging was performed preoperatively, and anterior cruciate ligaments (ACL) were confirmed to be intact. The mean age of the patients at the time of surgery was 73.6 ± 6.1 years (range, 54 to 87 years). The mean follow-up period was 36.5 ± 12.3 months (range, 24 to 63 months), and the follow-up rate was 95.8%. Demographic information, including age, sex, diagnosis, height, body weight, and preoperative passive knee flexion angle, was obtained from medical records. The patients were informed that data would be submitted for publication and gave their consent.

UKA was performed or assisted by 2 senior surgeons using a spacer block technique. The knees were exposed through a limited medial parapatellar approach. First, a proximal tibial cut was made slightly varus to the mechanical axis in the coronal plane using an extramedullary guide. The posterior inclination in the sagittal plane was set at 7° as recommended by the manufacturer.

A total of 2 pins were inserted during proximal tibial cut. One central pin for the proximal axis and one for fixing the cutting guide were temporarily inserted. Following the proximal tibial cut, a spacer block was inserted to measure the extension and flexion gap between the femoral articular surface and the tibial osteotomy surface. Thereafter, distal and posterior femoral resections were performed using the spacer block and a dependent cut technique [1]. If the flexion gap was tighter than the extension gap, the posterior femoral condyle was cut to be 1 or 2 mm thicker than the standard procedure. TRIBRID (Kyocera, Osaka, Japan), which is fixed-bearing UKA with a flat-surface polyethylene insert, was implanted in all knees, and all prostheses were fixed with cement.

**Figure 1:**
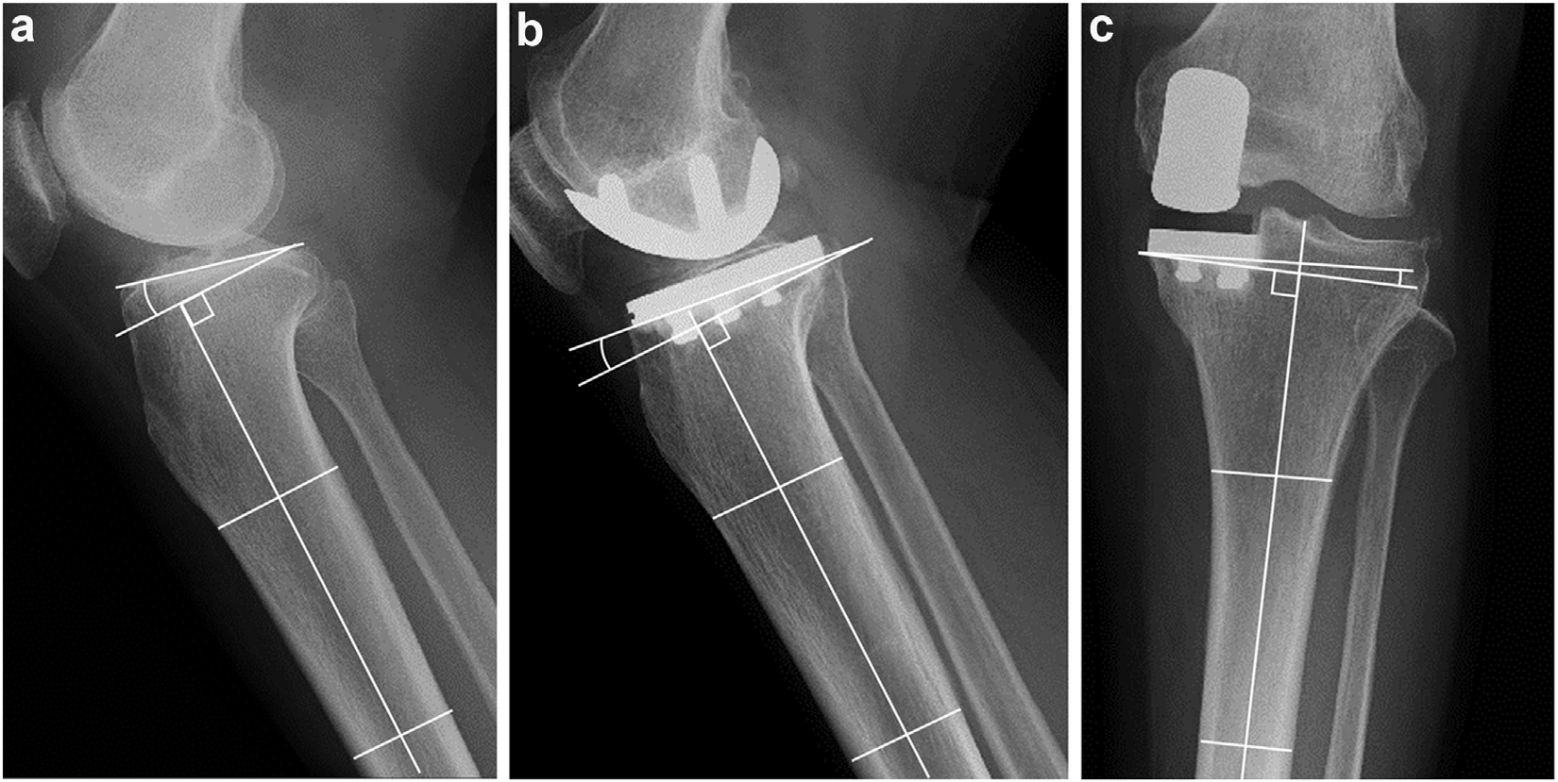
Radiographic measurement. Preoperative (a) and postoperative (b) posterior tibial slope and postoperative varus alignment of tibial component placement (c) were measured.

The preoperative and postoperative PTS were measured on lateral radiographs of the knee to 1 decimal place using an iRad-IA viewer (Infocom, Tokyo, Japan) [13,15]. The PTS reference line was defined as the line connecting the center of the medullary canal 7 to 15 cm distal to the tibial plateau. The preoperative PTS was defined as the angle between the perpendicular line of reference and a line connecting the anterior and posterior borders of the medial tibial plateau (Fig. 1a). The postoperative PTS was defined as the angle between the perpendicular line of reference and the undersurface of the tibial component (Fig. 1b). The postoperative reduction in PTS was also calculated. Knees were placed in the large reduction group if the postoperative PTS was reduced by more than 5° compared with the preoperative value or in the small reduction group if the postoperative PTS was not reduced by more than 5°. In addition, postoperative varus alignment of the tibial component placement (VATC) in the coronal plane was measured on postoperative anteroposterior radiographs of the knee to 1 decimal place. The VATC reference line was defined as the line connecting the center of the medullary canal 7 to 15 cm distal to the tibial plateau. The postoperative VATC was defined as the angle between the perpendicular line of reference and the undersurface of the tibial component (Fig. 1c). The hip-knee-ankle (HKA) angle, which was defined as the angle formed between the mechanical femoral axis and the mechanical tibial axis, was also measured on weight-bearing full-leg radiographs 1 year after surgery. The HKA angle was expressed as a deviation from 180° with a negative value for varus and positive value for valgus alignment. On postoperative lateral radiographs, if the anterior edge of the tibial component was in contact with the anterior cortex of the proximal tibia, it was evaluated that anterior cortical support was obtained. To evaluate the intraobserver and interobserver reliability, the preoperative and postoperative PTS, postoperative VATC, and postoperative HKA angle were measured in 2 independent trials on 20 randomly selected knees. The intraclass correlation coefficients of the preoperative and postoperative PTS, postoperative VATC, and postoperative HKA angle were 0.86, 0.92, 0.90, and 0.92, respectively, and the interclass correlation coefficients were 0.82, 0.89, 0.88, and 0.90, respectively.

Clinical and radiographic follow-up were completed at 3 weeks, 3 months, 6 months, 1 year, and subsequently every year after surgery. The range of motion values for all knees were measured using a goniometer, and clinical outcomes were measured using the 2011 Knee Society Knee Scoring System (KSS 2011) at the last follow-up [16].

All statistical analyses were performed using SPSS software (version 21.0; IBM, Armonk, NY). The Student’s t-test was used to compare age, height, body weight, body mass index, PTS, VATC, and HKA between the groups. The Mann-Whitney U-test was used to compare the preoperative and postoperative knee flexion angle, reduction in PTS, and postoperative KSS 2011. Spearman’s rank correlation coefficient was used to investigate the relationship between the preoperative and postoperative knee flexion angle. The χ^2^ test was used to compare sex distributions. Fisher’s exact test was used to compare the incidence of anterior collapse of the tibial component. A *P* value < .05 was considered significant. A post hoc power analysis using G∗Power 3.1 was used to determine the power in the comparison of collapse occurrence rates between the large reduction group and the small reduction group. With an underlying α of 0.05 and a sample size of 182 knees, a power of 0.816 was calculated.

## Results

**Figure 2:**
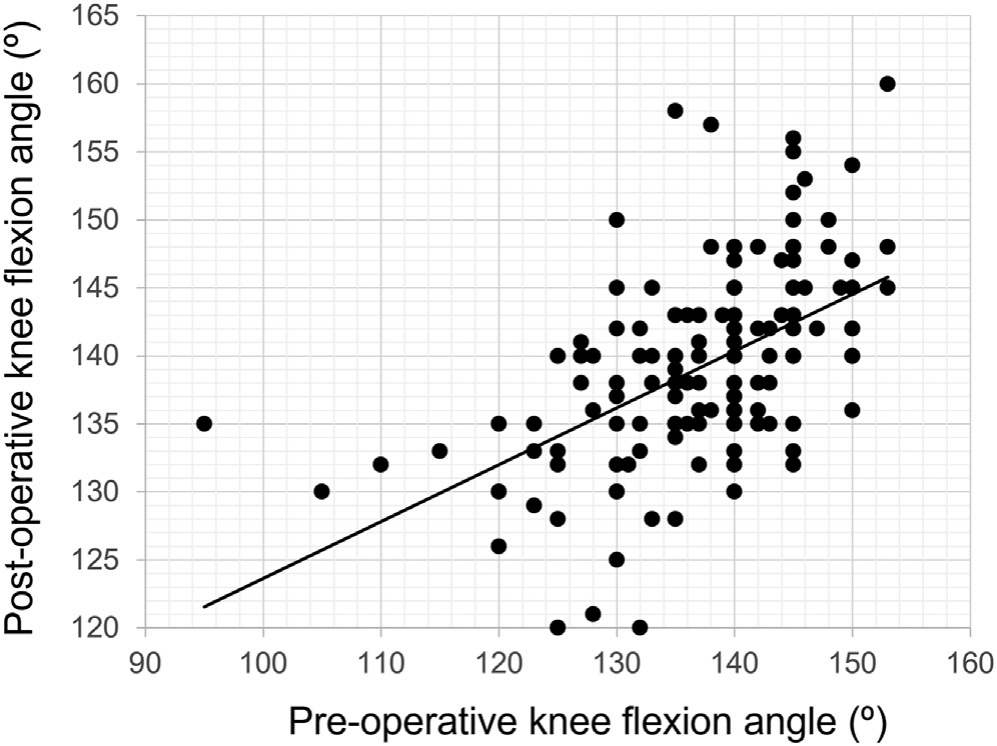
Correlation between preoperative and postoperative knee flexion angles. There was a significant positive correlation (r = 0.53, *P* < .001).

The mean preoperative and postoperative PTS values were 8.3 ± 2.9° and 6.5 ± 2.7°, respectively. Fifty-eight knees (31.9%) had a preoperative PTS of greater than 10°. The mean preoperative knee extension and flexion angle were −3.6 ± 3.9° and 137.9 ± 9.0°, respectively. The mean knee extension and flexion angles at the last follow-up were −0.6 ± 1.8° and 139.4 ± 7.1°, respectively. There was a significant positive correlation between the preoperative and postoperative knee flexion angles (r = 0.53, *P* < .001) (Fig. 2).

There were 33 knees in the large reduction group and 149 knees in the small reduction group (Table 1). Preoperative PTS in the large reduction group was significantly larger than that in the small reduction group (*P* < .001, Table 1). Furthermore, the postoperative PTS in the large reduction group was significantly smaller than that in the small reduction group (*P* < .001, Table 1). Postoperative VATC and HKA were not significantly different between the groups (Table 1). At the last follow-up, the knee flexion angle and KSS 2011, including postoperative pain on level walking and on stairs, satisfaction, expectation, and functional activity, were not significantly different between the groups (Table 2).

**Table 1:** Demographic data.

	Large reduction group (N = 33)	Small reduction group (N = 149)	*P* value
Age (y)	73.3 [58, 82]	73.6 [54, 87]	.818
Sex (male/female)	11/22	56/93	.647
Diagnosis (OA/ON)	20/13	110/39	.128
Height (cm)	156.5 [144, 175]	154.9 [138, 176]	.326
Body weight (kg)	63.9 [46, 87]	62.8 [42, 104]	.596
BMI (kg/cm^2^)	26.1 [19.9, 34.9]	26.1 [19.0, 39.2]	.981
Preoperative knee flexion angle (°)	135.5 [105, 153]	138.4 [95, 153]	.096
Preoperative PTS (°)	10.9 [0, 14.9]	7.7 [−1.1, 14.0]	<.001
Postoperative PTS (°)	3.6 [−2.5, 8.0]	7.1 [−0.3, 14.6]	<.001
Postoperative VATC (°)	4.1 [−1.6, 9.1]	4.1 [−1.8, 11.1]	.903
Postoperative HKA (°)	−4.1 [−9.5, 2.4]	−4.4 [−11.3, 2.9]	.666

BMI, body mass index; OA, osteoarthritis; ON, osteonecrosis; VATC, varus alignment of tibial component placement.

Data are expressed as median [minimum, maximum].

**Table 2:** Comparison of clinical outcomes at the last follow-up between the large reduction group and small reduction group.

	Large reduction group (N = 33)	Small reduction group (N = 149)	*P* value
Postoperative knee flexion angle (°)	138.3 [116, 160]	139.7 [120, 158]	.243
KSS 2011			
Symptom (point)	20.2 [4, 25]	20.8 [2, 25]	.542
Pain on level walking	1.3 [0, 8]	1.0 [0, 8]	.337
Pain on stairs or incline	2.1 [0, 8]	1.5 [0, 10]	.121
Satisfaction (point)	27.6 [12, 40]	27.6 [10, 40]	.643
Expectation (point)	8.8 [3, 15]	9.7 [3, 25]	.181
Functional activity			
Walking and standing (point)	21.1 [6, 30]	23.2 [0, 30]	.180
Standard activities (point)	24.7 [11, 30]	25.0 [6, 30]	.413
Advanced activities (point)	15.6 [0, 25]	15.1 [0, 25]	.963
Discretionary activities (point)	9.9 [1, 15]	9.8 [0, 15]	.727
Number of knees with anterior collapse of the tibial component (knees)	4 (12.1%)	1 (0.7%)	<.001

Data are expressed as the mean [minimum, maximum] or the number (percentage).

**Figure 3:**
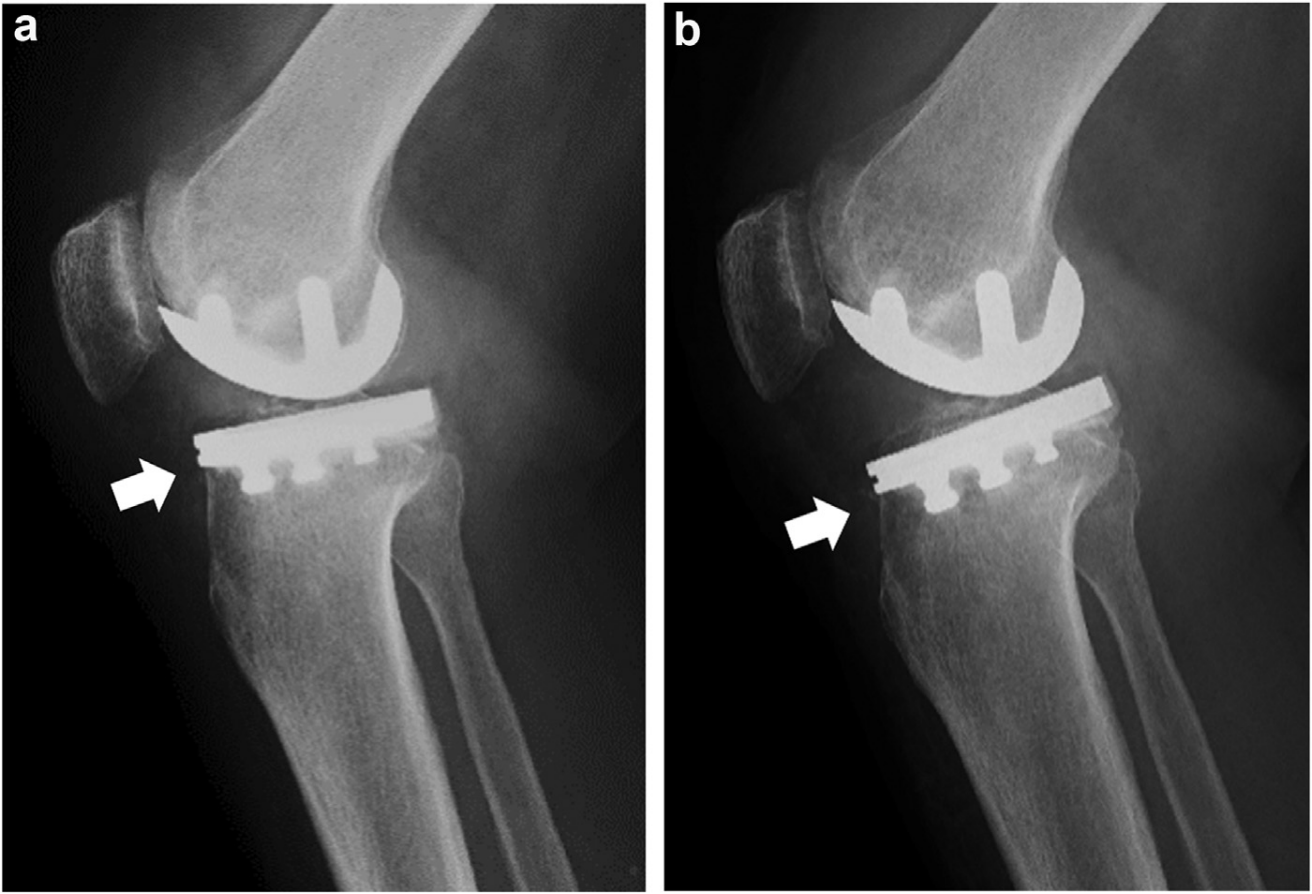
Case 4, 70-year-old female. (a) Postoperative radiography. Anterior tibial cortical support was achieved (arrow). (b) Three months after surgery, anterior collapse of the tibial component was observed (arrow).

**Figure 4:**
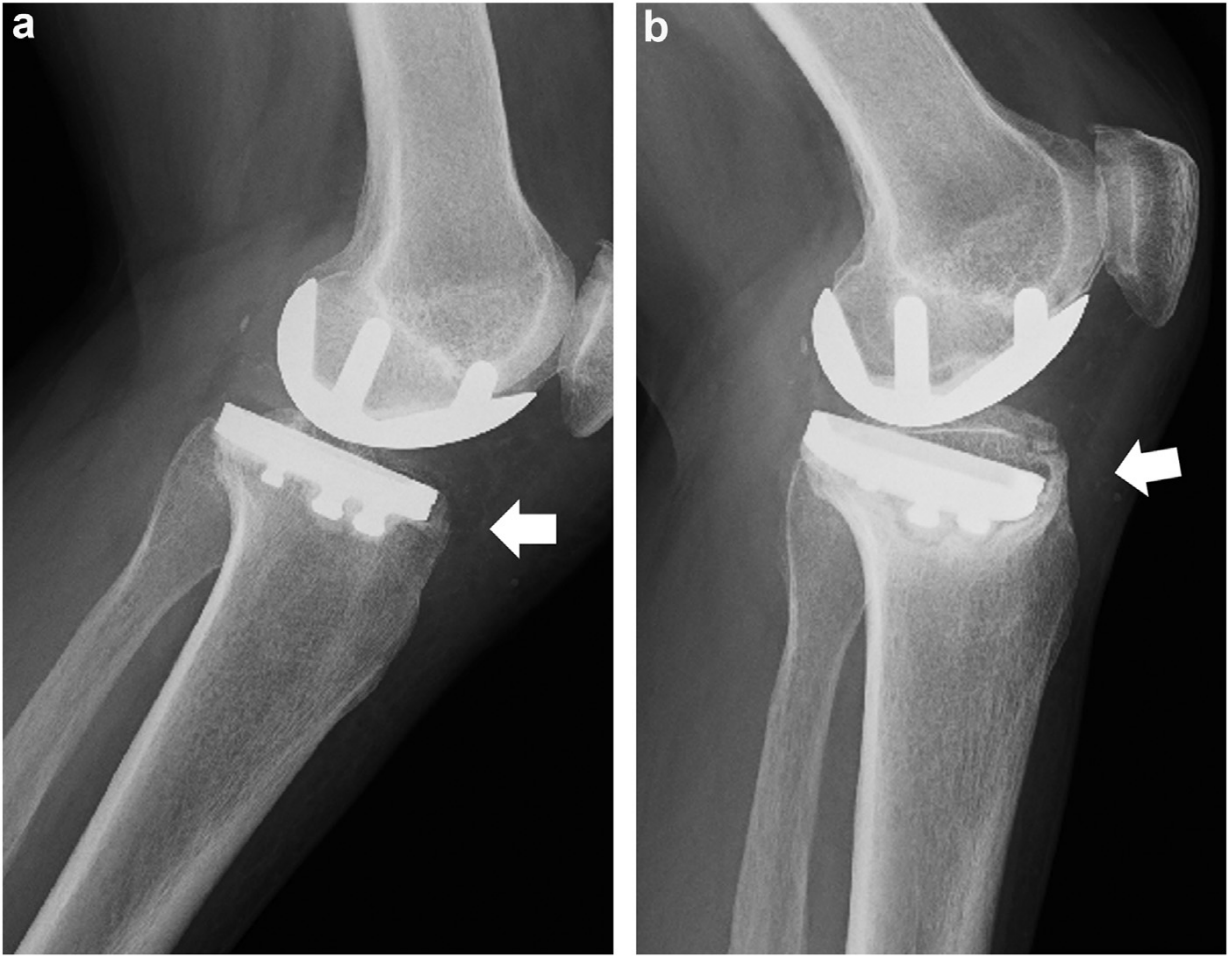
Case 2, 67-year-old female. (a) Postoperative radiography. Anterior tibial cortical support was not achieved (arrow). (b) Eleven months after surgery, collapse of the tibial component progressed (arrow).

By the last follow-up visit, 5 knees (2.7%) exhibited anterior collapse of the tibial component (Fig. 3a and b, Fig. 4a and b, Table 3). Of 5 cases of anterior collapse, 4 cases were in the large reduction group, and 1 case was in the small reduction group. There were no patients with posterior collapse. Two knees exhibited anterior collapse on lateral radiographs 3 weeks after surgery, and 3 knees were observed 3 months after surgery. All patients were female, and the mean age at surgery was 72.6 years (range, 65 to 82 years). Anterior cortical support in placement of the tibial component on the tibial cut surface was confirmed in 3 of 5 knees on postoperative radiography (Fig. 3a). Four patients did not undergo revision surgery because bone union was achieved, and pain decreased after conservative treatment using crutches to avoid weight-bearing on the affected limb. One patient (case 2) underwent conversion TKA 11 months after UKA because collapse of the tibial component progressed, and pain was constant. Anterior tibial cortical support was not achieved (Fig. 4a and b). The values of reduction in PTS of the knees with anterior collapse of the tibial component were significantly higher than those of knees without it (*P* = .01, Table 4) although the postoperative PTS was not significantly different between groups. Also, the incidence of anterior collapse of the tibial component in the large reduction group was significantly higher than that in the small reduction group (*P* < .001, Table 2).

**Table 3:** Cases of anterior collapse of the tibial component.

Case	Age (y)	Sex	Diag.	BMI (kg/cm^2^)	Preoperative PTS (°)	Postoperative PTS (°)	Reduction in PTS (°)	Postoperative VATC (°)	Postoperative HKA (°)	Date identified on radiograph	Conversion to TKA
1	65	F	OA	28.6	14.0	2.6	11.4	1.9	0	3 wk to 3 mo	No
2	67	F	ON	27.3	10	11.1	−1.1	7.5	N/A	Within 3 wk	Yes (11 mo postoperatively)
3	79	F	ON	25.9	10.4	3.3	7.1	8.4	−9.4	Within 3 wk	No
4	70	F	OA	29.3	13.7	6.8	6.9	4.8	−9.5	3 wk to 3 mo	No
5	82	F	OA	26.4	11.5	5.9	5.6	5.9	−4.3	3 wk to 3 mo	No

BMI, body mass index; F, female; N/A, not applicable; OA, osteoarthritis; ON, osteonecrosis.

**Table 4:** Comparison between the knees with anterior collapse and those without.

	Anterior collapse (N = 5)	No collapse (N = 177)	*P* value
Age (y)	72.6 [65, 82]	73.6 [54, 87]	.724
Preoperative PTS (°)	11.5 [10, 14]	7.9 [−1.1, 15.5]	.005
Postoperative PTS (°)	5.9 [2.6, 11.1]	6.5 [−2.5, 14.6]	.653
Reduction in PTS (°)	6.0 [1.1, 11.4]	1.6 [−8.1, 12.6]	.028
Preoperative HKA (°)	−4.7 [−10.3, 1.6]	−8.0 [−21.6, 2.5]	.403
Postoperative VATC (°)	84.3 [81.6, 88.1]	85.9 [78.9, 91.8]	.146
Postoperative HKA (°)	−5.8 [−9.5, 0]	−4.3 [−11.3, 2.9]	.398

Data are expressed as the mean [minimum, maximum].

## Discussion

In the present study, the knee flexion angle and KSS 2011 at the last follow-up were not significantly different between the large reduction group and the small reduction group. These results mean that our initial hypothesis is not supported. However, the incidence of anterior collapse of the tibial component in the large reduction group was significantly higher than that in the small reduction group.

Many surgeons routinely aim for a PTS of 5° to 7°. Hernigou and Deschamps [12] reported that 5 of 81 knees that had had a normal ACL at the time of UKA had no ACL at the time of revision surgery, suggesting that disruption of the ACL occurred in relation to a greater posterior slope (>10°). However, 1 of the limitations of their study was that the preoperative and postoperative changes in PTS were unknown. Failures attributed to a large PTS may have occurred in patients with minimal PTS preoperatively that significantly increased after undergoing UKA, causing abnormal stress on the ACL. There are wide variations in the preoperative PTS, and nearly half of all preoperative tibial slopes are >7° [14]. In particular, Asian patients were reported to have a higher PTS than Western patients [17,18]. Therefore, a routine PTS target of 5° to 7° may fail to recreate the native anatomy in a large percentage of patients.

A postoperative decrease in PTS after TKA was reported to result in a tight flexion gap [13,19]. In cruciate-retaining TKA, the influence on the flexion gap caused by changing the PTS by 5° was reported to be approximately 2 mm [19]. A tight flexion gap leads to loss of knee flexion. In the present study, a decrease in PTS of more than 5° did not result in loss of the knee flexion angle after UKA. UKA in this series was performed using a spacer block technique. If the flexion gap was tighter than the extension gap due to a smaller PTS than the native slope, the posterior femoral condyle cut was made 1 or 2 mm thicker than the standard procedure, thereby widening the flexion gap. This procedure may prevent a decrease in the knee flexion angle, and there was a significant positive correlation between the preoperative and postoperative knee flexion angles (Fig. 2). There are no previous reports that compared preoperative and postoperative PTS change and PROMs in UKA. A previous study [20] which evaluated the effect of postoperative PTS in UKA on clinical outcomes and knee flexion angle in UKA reported that postoperative knee flexion angle was significantly larger in large postoperative PTS groups than that in the small postoperative PTS group, but no difference was observed in PROMs. In our study, there is no difference in PROMs between the groups, and it is expected that the effect of PTS change on PROMs is small, at least in the midterm follow-up.

The major problem with UKA is the lower survival rate than that with TKA. Documented complications related to UKA include disease progression on lateral side, tibial collapse and loosening, and bearing dislocation [21,22]. Medial tibial collapse was reported as a main cause of UKA early failure [21]. In contrast to TKA, the interface between the tibial component and tibial bone is significantly smaller. This suggests that the underlining bone stresses are more sensitive to component malalignment. The cause of medial tibial collapse after UKA was multifactorial, including component alignment, poor bone quality, female sex, and obesity [11,23]. Cortical coverage is recommended for the prevention of collapse of the tibial component [24]. In the present study, anterior cortical support was not achieved in 2 of 5 collapsed knees in all 182 cases, and 1 patient underwent conversion TKA 11 months after UKA. We considered surgical error to be a cause of collapse in these knees. In addition, the postoperative PTS was as small as 3.6° on average in the large reduction group. This point may also be considered as a surgical error because it is smaller than the actual target of 7°.

In contrast, in 3 of 5 collapsed knees, although anterior cortical coverage was confirmed on postoperative radiography (Fig. 3a), anterior collapse of the tibial component was noted (Fig. 3b). The postoperative PTS of these 3 knees was reduced by more than 5° compared with the preoperative value. To date, no study has investigated the influence of a large reduction in PTS after UKA compared with preoperative PTS on knee kinematics or anterior and anteromedial strain. In the present study, anterior collapse of the tibial component occurred in 4 of 33 knees (12.1%) in the large reduction group, and the incidence of anterior collapse in the large reduction group was significantly higher than that in the small reduction group. Furthermore, the values of reduction in PTS of the knees with anterior collapse of the tibial component were significantly higher than those of knees without. A proximal tibial cut with a smaller PTS than the preoperative PTS results in a larger anterior tibial bone cut than the posterior tibial bone cut. We infer anterior collapse of the tibia component may be caused by inferior bone quality of the anterior bone cutting surface due to the larger anterior bone cut. Individualized targeting for PTS may be better for patients undergoing UKA in order to avoid anterior collapse of the tibial component. On the other hand, there is a limit to the amount of PTS that can be adjusted by the tibial cut guide provided by the standard manufacturer, and it is difficult to reproduce the native PTS in patients with a large preoperative PTS of 10 degrees or more. Computer assist such as robotically assisted or navigated UKA may be advantageous because there is a possibility that the target PTS can be reproduced more accurately than the manual standard tibial guide. Longer follow-up is required to clarify whether progressive disruption of the ACL occurs over time when the tibial component is placed with a greater PTS to reproduce the preoperative native PTS.

This study has several limitations. First, this is a retrospective study, and the causality is unknown. Therefore, prospective randomized trials are needed. Second, we had a relatively small sample size. Third, all subjects were Japanese. Therefore, caution should be used when applying these findings to patients of different ethnicity. Fourth, the follow-up period was relatively short. However, anterior collapse occurred within 3 months of UKA, and it was not observed 6 months after surgery. Fifth, the results of the present study are implant-specific because a single product was used in our study. Therefore, the generalizability may not be present. Sixth, the bone mineral density was not evaluated. In particular, the diagnosis or medication of osteoporosis in all patients could not be recorded. Seventh, the incidence of collapse was evaluated by only follow-up radiography. Eighth, postoperative full-leg radiographs before collapse were not obtained. All knees achieved weight-bearing 1 year after surgery based on full-leg radiography, but anterior collapse occurred within 1 year after UKA. Although coronal placement of the tibia component was evaluated on short films of the knee after surgery, we were not able to determine the relationship between coronal long-leg alignment and collapse. Ninth, there is no standard in the literature on how PTS should be measured on radiographs. Therefore, we measured with reference to previous reports [13,15]. Tenth, since operations were performed manually rather than robotically, postoperative PTS actually varied despite being targeted at 7°, which implies a lack of accuracy.

## Conclusions

Although the knee flexion angle and PROMs at the last follow-up were not significantly different between the groups, a large reduction in postoperative tibial slope may be associated with anterior tibial collapse. Therefore, this study shows one potential benefit for matching native slope.

## Conflict of interest

The authors declare there are no conflicts of interest.

For full disclosure statements, refer to https://doi.org/10.1016/j.artd.2022.06.011.

## Appendix A Supplementary Data

Conflict of Interest Statement for Higuchi

Conflict of Interest Statement for Saito

Conflict of Interest Statement for Terauchi

Conflict of Interest Statement for Chikuda

Conflict of Interest Statement for Kurihara

Conflict of Interest Statement for Hatayama
